# VarBin, a novel method for classifying true and false positive variants in NGS data

**DOI:** 10.1186/1471-2105-14-S13-S2

**Published:** 2013-10-01

**Authors:** Jacob Durtschi, Rebecca L Margraf, Emily M Coonrod, Kalyan C Mallempati, Karl V Voelkerding

**Affiliations:** 1 ARUP Institute for Clinical & Experimental Pathology®, Salt Lake City, Utah, USA; 2 Department of Pathology, University of Utah School of Medicine, Salt Lake City, Utah, USA

## Abstract

**Background:**

Variant discovery for rare genetic diseases using Illumina genome or exome sequencing involves screening of up to millions of variants to find only the one or few causative variant(s). Sequencing or alignment errors create "false positive" variants, which are often retained in the variant screening process. Methods to remove false positive variants often retain many false positive variants. This report presents VarBin, a method to prioritize variants based on a false positive variant likelihood prediction.

**Methods:**

VarBin uses the Genome Analysis Toolkit variant calling software to calculate the variant-to-wild type genotype likelihood ratio at each variant change and position divided by read depth. The resulting Phred-scaled, likelihood-ratio by depth (PLRD) was used to segregate variants into 4 Bins with Bin 1 variants most likely true and Bin 4 most likely false positive. PLRD values were calculated for a proband of interest and 41 additional Illumina HiSeq, exome and whole genome samples (proband's family or unrelated samples). At variant sites without apparent sequencing or alignment error, wild type/non-variant calls cluster near -3 PLRD and variant calls typically cluster above 10 PLRD. Sites with systematic variant calling problems (evident by variant quality scores and biases as well as displayed on the iGV viewer) tend to have higher and more variable wild type/non-variant PLRD values. Depending on the separation of a proband's variant PLRD value from the cluster of wild type/non-variant PLRD values for background samples at the same variant change and position, the VarBin method's classification is assigned to each proband variant (Bin 1 to Bin 4).

**Results:**

To assess VarBin performance, Sanger sequencing was performed on 98 variants in the proband and background samples. True variants were confirmed in 97% of Bin 1 variants, 30% of Bin 2, and 0% of Bin 3/Bin 4.

**Conclusions:**

These data indicate that VarBin correctly classifies the majority of true variants as Bin 1 and Bin 3/4 contained only false positive variants. The "uncertain" Bin 2 contained both true and false positive variants. Future work will further differentiate the variants in Bin 2.

## Background

Next Generation Sequencing (NGS) of whole genomes or exomes has been a transformational tool for discovering causal variants in human diseases and uncovering new relationships between genes and disease mechanisms. Sequencing the exome or genome yields thousands to millions of variant changes from a human reference sequence, ranging from a single nucleotide variant to more complex variants (such as insertions and deletions). Analysis and identification of causal variants in these large data sets can be difficult due to the presence of many false positive variants that are commonly due to sequencing chemistry errors [1,2] and/or alignment errors. Differentiating between true variants and false positive variants is an outstanding challenge in causal variant discovery efforts and a robust method for prioritization of true variants over false positive variants would decrease analysis time and increase confidence in the results of variant discovery projects. This requires a balance between removal of false positive variants (specificity) while retaining true variants (sensitivity) [3].

Sequencing accuracy of Illumina's NGS instruments have improved over time with the majority of sequencing chemistry errors being base substitution errors [1]. Although base substitution error rates are on average relatively low (less than 1%), sequence-specific errors occur at higher rates [4-7]. These sequence-specific error sources include homopolymer tracks that are often followed by an error matching the homopolymer base. Homopolymers as short as two nucleotides may cause increased sequencing error at the next nucleotide. Particularly GGT and GGA sequence motifs commonly lead to a miscall of the last base as a G [6]. However, these known problematic sequences do not always coincide with increased sequencing error rates, and cannot be relied on alone to confirm or disprove an error. Alignment errors, typically associated with repetitive or homologous sequence regions, are also a source for false positive variant calls and can lead to single to several base substitutions, insertion, and deletion errors [8,9]. Since both sequencing errors and alignment errors can be associated with certain sequence motifs, these errors can be consistent between samples sequenced on the same instrument using the same sequencing chemistry and alignment methods, as demonstrated in several studies [4,10-13].

Basic alignment and variant calling parameters within the Genome Analysis Tool Kit (GATK) and other variant calling software have been developed to help identify and reduce false positive variants [14,15]. These include the probabilistic base quality and alignment mapping quality score, the aligned read coverage for possible alleles, and, more recently, the base alignment quality score [16]. These parameters can be used to apply variant hard filters but are more often used in a statistical model to generate a probabilistic, Phred-scaled, variant quality score (QUAL). This score is an estimated probability of a false positive variant but can be incorrect at sites prone to sequencing and/or alignment errors. To identify and address inherent error in the QUAL score, additional alignment parameters have been derived including several bias values that compare reference matching reads versus variant containing reads for systematic differences in base quality, mapping quality, mapping position, and mapping direction. The simplest use of these parameters is to screen variants using a set of filter values chosen for each bias parameter. A more sophisticated implementation is employed in the GATK software as variant quality score recalibration (VQSR) in which a multidimensional Gaussian mixture model is trained and then used to infer variant truth likelihood based on each variant's parameter combination [14]. VQSR evaluates variants from many different sites across the entire sequence area simultaneously but because the characteristics of error from site to site can differ and are difficult to characterize, site specific issues are not fully captured by the current set of parameters used in the VQSR method. Other methods incorporate additional information to more accurately identify error prone positions. These include methods for detection of false positive variants around repetitive or homologous sequence [17,18], or methods that utilize cross-sample error information by requiring multiple background samples [10,13,19]. These cross-sample methods evaluate characteristics of reads from multiple samples that likely contain similar errors at the variant site of interest.

This report presents VarBin, a novel analytical method for classifying variants as true or false positive in Illumina NGS data. VarBin is a variant likelihood binning method for heterozygous variants of types including single nucleotide variants (SNVs), insertion, and deletion variants, as well as variants in homopolymer and repeat regions. The VarBin method evaluates each variant site individually to focus on site-specific alignment information for false positive variant determination. Also, this method uses multiple background samples to take advantage of the cross-sample error characteristics that show similar trends between samples sequenced on the same platform with the same chemistry and alignment method. VarBin uses Genotype likelihood scores (PL) to generate PLRD (the variant likelihood ratio over coverage depth), a value affected by alignment and sequencing error. VarBin compares the PLRD calculated for the proband variant to those calculated for multiple background samples for each variant change and position.. This report describes the VarBin method and its performance characteristics.

## Methods

### Whole genome or exome next-generation sequencing

One family that was Illumina whole genome sequenced was chosen as the test case for analysis and consists of a male proband and his unaffected mother, father, and brother. An additional 38 samples were either whole genome (8 samples) or exome sequenced (30 samples) and used as the background NGS data for analysis. This set of samples consists of 8 family groups (3 to 5 individuals per family) as well as 4 unrelated individuals. Each family was unrelated to each other (or the test family) Sequencing data used for this study were generated in studies approved by the University of Utah Institutional Review Board.

For each family member, 2.5 ug of genomic DNA was fragmented to a 300 - 400 bp size distribution, using the Covaris™ instrument (Covaris, Inc., Woburn, MA, USA). Illumina specific libraries were generated using the automated SPRI-TE instrument (SPRI-TETM Nucleic acid extractor, Beckman Coulter Genomics, Danvers, MA, USA), then amplified using Illumina PCR primers 1.0 and 2.0. For the exome libraries only, the in solution capture was performed according to the Roche Nimblegen SeqCap EZ Human Exome Library v2.0 or v3.0 (Madison, Wisconsin) instructions with the following exceptions. The adapter ligated libraries were PCR amplified prior to probe hybridization by splitting the library volume across eight individual PCR tubes each with a 50 μl total volume. Following PCR, the reactions were pooled and purified according to the manufacturer's instructions and subsequently used for probe hybridization. Genome or exome libraries were then gel purified in the range of 475 +/- 50 bp and the library concentrations were determined using qPCR (KAPA Library Quant Kit, Kapa Biosystems, Inc., Woburn, MA, USA). The Illumina cBot instrument was used for cluster generation, followed by sequencing on the HiSeqTM 2000 instrument with 100 base length paired-end reads.

### NGS alignments and variant calling

FastQ file sequencing reads for each sample were aligned using the BWA aligner (version 0.6.1, default settings for paired ends). This initial alignment was followed by local realignment around indels (GATK version 1.5) [14,20]. The preliminary variant calls and dbSNP 132 were used to identify potential sites for local realignment. Duplicate read removal was performed using Samtools (rmdup option, default settings). Base quality recalibration was performed with GATK using known variant sites identified by dbSNP 132 and the variant calls from the initial alignment. Base quality covariates used were base quality score, cycle number, and the proceeding dinucleotide. The final alignment files (bam format) were used to make initial variant calls in the vcf file format (GATK UnifiedGenotyper tool) with only the following non-default settings. Maximum coverage was set to 1000, stand_call_conf was 30, std_emit_conf was 10 and base alignment quality option (-baq) was set to CALCULATE_AS_NECESSARY. Variant quality score recalibration (VQSR) in GATK was used to update the vcf files with estimated false positive likelihood odds ratios (VQSLOD). Optional variant hard filter values, defined in the GATK best practices, were applied (VariantFiltration tool in GATK) for SNVs as QD < 2.0, MQ < 40.0, FS > 60.0, HaplotypeScore > 13.0, MQRankSum < -12.5, or ReadPosRankSum < -8.0 and flagged in the filter fields as failing. Similarly, Indel hard filter limits were QD < 2.0, ReadPosRankSum < -20.0, or FS > 200.0. Only the preliminary called variants that passed these hard filters were considered a variant in the vcf file, while those that did not pass these variant calling filters are termed non-variants. These optional hard filters were used to remove many of the lowest quality variants.

### VarBin: variant heterozygous likelihood binning method

The phred-scaled, genotype likelihood values for the possible genotypes, PL(AA) (homozygous wild type), PL(BB) (homozygous variant), and PL(AB) (heterozygous variant) are found in the GATK UnifiedGentyper vcf file, are derived from mapping, alignment, and base qualities, allele read percentage, and read coverage at a given putative variant site. These PL values tend to show erroneously increased variant likelihoods at positions prone to sequencing and alignment errors. VarBin takes advantage of this systematic error in the PL values as an indicator of false positive variant calls, by first calculating a phred-scaled, genotype likelihood ratio (PLR),

(1) $$\text{PLR}=-10\cdot {\text{log}}_{10}\left(\frac{\text{PL}{\left(\text{AA}\right)}_{linear}}{\text{PL}{\left(\text{AB}\right)}_{linear}+\text{PL}{\left(\text{BB}\right)}_{linear}}\right)$$

where PL(AA)linear, PL(AB)linear, and PL(BB)linear are PL, converted from a Phred to a linear scale. To focus on the effects of alignment and sequencing error in PLR, its strong linear correlation to read coverage depth is minimized through conversion to a more coverage-independent parameter, PLR by depth (PLRD)

(2) $$\text{PLRD}=\frac{\text{PLR}}{\text{DP}}$$

where DP is the raw, read coverage depth (from the vcf file). The PLRD value for each proband variant of interest was then compared to PLRD values for the same variant change and position from each background sample alignment (bam file).

To generate the needed data for these PLRD calculations and comparisons, the GATK UnifiedGenotyper was employed. Nonstandard UnifiedGenotyper settings were needed to force vcf file values to be created at "non-variant" sites (either wild type at that position or a variant call that did not pass filters). The genotype_likelihoods_model was set to BOTH. The stand_call_conf was set to 0.0. The stand_emit_conf option was set to 0.0. The max_deletion_fraction was set to 1.0. The min_base_quality_score option was set to 17. The genotyping_mode option was set to GENOTYPE_GIVEN_ALLELES and the associated alleles option was set using a variant file (vcf format) containing the variants of interest that had previously been identified in the proband sample. The output_mode option was set to EMIT_ALL_SITES.

The resulting single proband and multiple background vcf files each contained entries for the same set of variant changes and positions of interest. Variant filters defined in the GATK recommended best practices and listed above were then applied to the background samplevariants. Note that this filtration process provided an imperfect but useful estimated separation of wilt type/non-variants from true variants. Variants not passing these filters were marked as wild type/non-variant in the vcf file filter field. Vcftools (version 0.1.8, http://vcftools.sourceforge.net) was then used to extract pertinent data from the proband vcf file and all background sample vcf files (filter field data, DP, and PL values).

Each variant of interest was analyzed independently of all others. First, the distribution of PLRD values from all background samples for the same variant change and position, that were called wild type/non-variants, as stated in the vcf file, was analyzed. This included calculation of the medians and inter-quartile distances (IQD). A 1.38*IQD was used as a proxy for one standard deviation in a Gaussian distribution.

This study focused on the heterozygous variants, which are more likely to contain false positives variants than homozygous calls (due to the overall number of variant containing reads). Homozygous variants were excluded from further analysis, but were given a PLRD value of zero for plotting purposes only. A variant was considered homozygous if PL(BB) was larger than PL(AA) or PL(AB).

For VarBin analysis of each variant of interest, the proband PLRD value was compared to the background sample PLRD distribution values and classified or "binned" based on a heuristic that was an automation of the original manual visual interpretation of alignment data as an indicator of sequencing or alignment error. Bin 1 includes proband variants that have a PLRD value greater than 10 and greater than the distribution median plus 6 proxy standard deviations (8.28*IQD). Bin 2 includes proband variants with a PLRD value greater than 10 or greater than the distribution median plus 6 proxy standard deviations but not both. Bin 3 contains variants with a PLRD value less than the distribution median plus 8.28*IQD but greater than the distribution median plus 3 proxy standard deviations (4.14*IQD). Bin 4 contains variants with a PLRD value less than the distribution median plus 3 proxy standard deviations. The likelihood of a true variant is highest in Bin 1 and lowest in Bin 4.

### Cross-sample annotation methods

In addition to VarBin's primary binning procedure, other valuable information about proband variant accuracy is gained from the cross-sample comparison with multiple background samples. Multiple parameters for each variant change and position were tracked in the proband, family members, as well as in the background files. These included lists of which background files had the corresponding variant call and zygosity, low read coverage depth (may indicate a no-call versus a wild type call for a family member), high strand bias, high base quality bias, mapping quality bias, and base position bias. These parameters helped track inheritance patterns within the family as well as trends that track with false positive variants.

### Generation of variant test sets

The test family was analyzed for rare and *de novo *variants within the proband. Proband variants were found by this screening method: >3 total read coverage depth, <3% minor allele frequency (MAF) in the 1000 Genomes, <2% MAF in the ESP5400 exomes, and not present in the unaffected family members (mother, father, and brother). In addition, since the 1000 Genomes and ESP5400 data set did not have insertions or deletions at the time of the study, variants that were found to be very common within several NGS datasets (present in greater than 3 unrelated families with different disorders) were eliminated as being too common of a variant for a rare disorder.

Additional proband variant test sets were generated to evaluate the VarBin method. One variant set was enriched for true variants (20,000 variants), by selection of only the proband's variants present between 10 and 20% MAF in the 1000 Genomes data within Chromosome 1. The other variant set was enriched for false positive variants (14,500 variants), by selection of only the novel and *de novo *proband variants (not present in the 1000 Genomes data and not found in family members) within chromosomes 1 through 22.

### Sanger sequencing

Big-Dye terminator Sanger sequencing was performed for NGS detected variant verification on a total of 98 variants to test the VarBin method's accuracy. One dataset was randomly selected from the proband's *de novo *variants found in or near coding regions within chromosome 1 through 6 (66 variant sites, 4 could not be Sanger sequenced). Another variant set was selected based on the proband's *de novo *variants. An additional 5 more Bin 1 proband variants in other chromosomes were sequenced. Eight Sanger sequenced proband variants were found in Bin 1 or 2 in a parent but was called wild type/non-variant in the parent's vcf file (potential false negatives) and were also Sanger sequenced. To expand the study to other families, 19 variants were sequenced in unrelated families that were in Bin 1 and 2 that were previously thought to be true variants by manual visual verification of the read data in iGV (Broad Institute).

## Results

### VarBin method

**Figure 1:**
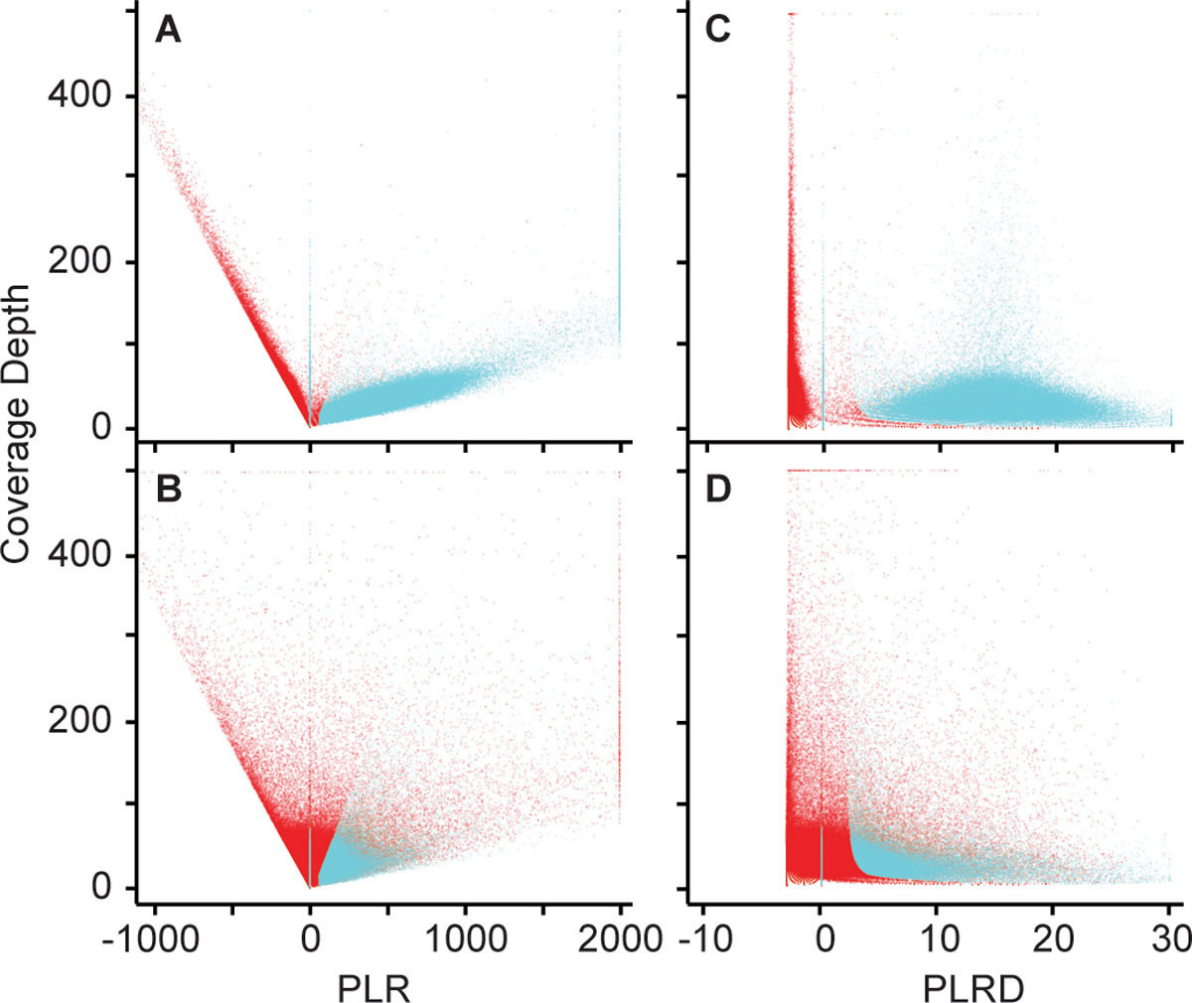
**Read coverage depth effect on likelihood scores**. Each data point represents one variant change per one sample from the study family's genomes and 38 background data files. Blue data points passed the GATK best practice variant filters and are called variants in the vcf file. Red data points are "non-variants" which did not pass these same filters. Homozygous variants were given an × axis value of zero to separate them from the heterozygous variant distributions. Values beyond the plot axis limits are shown at the plot edges. A and B) Scatter plot of the Phred-scaled likelihood ratio (PLR) versus coverage depth for two variant sets. A) Variant data set enriched for true variants. For this plot, variants in the study family proband within chromosome 1 were limited to between 10 and 20% allele frequency in 1000 Genomes data (20,000 variants total). B) Variant data set enriched for false positive variants. For this plot variants in the study family proband at chromosomes 1 - 22, then limited to variants not found in the 1000 Genomes data or either parent (14,500 variants total). C and D) Scatter plot of the Phred-scaled likelihood ratio divided by coverage depth (PLRD) versus coverage depth for two variant sets. C) Data from panel A divided by coverage depth. D) Data from panel B divided by coverage depth

Whole genome sequencing data from the test family included in this study was comprised a male proband and his unaffected father, mother, and brother. In addition, 38 unrelated background samples sequenced with the same instrument, sequencing chemistry, and alignment methods were used for analysis. Illumina errors tend to be consistent between samples using the same chemistry and instrument [10,13,19], and the background data were used to help identify these errors. By a visual and manual cross comparison of NGS read data in the Integrative Genomics Viewer (iGV) [21], it was possible to make predictions of likely true variants or false positive variants. This method was time consuming and difficult to analyze multiple background samples at once. To address this, the VarBin method was developed to provide a more automated and discriminating procedure for classifying true variants versus false positive variants. VarBin uses the likelihoods for the different possible genotypes (PL) that are generated as part of the statistical framework of variant detection in GATK and Samtools (see Methods). The PL values for wild type, heterozygous and homozygous genotypes at a variant position are combined into a variant Phred-scaled likelihood ratio (PLR) that is strongly affected by read coverage depth (Figure 1A and 1B). To demonstrate the correlation of PLR with depth, a set of proband variant calls enriched for true variants was created (20,000 variants, Figure 1A) and a data set enriched for false positive variants was created (14,500 variants, Figure 1B). These two data sets were used to plot PLR versus depth for the proband variant calls as well as all PLR values within background data sets corresponding to the same variant change and position as for the proband. Variants are plotted to the left and right of zero on the PLR × axis depending on their relative likelihood to be a false positive variant or a true variant, respectively. A linear distribution of PLR versus coverage depth was observed for data points in Figure 1A and 1B. PLR was divided by corresponding coverage depth (DP) to derive a more coverage independent parameter: PLR by depth (PLRD). Minimal effect of coverage on PLRD is demonstrated (Figure 1C and 1D).

**Figure 2:**
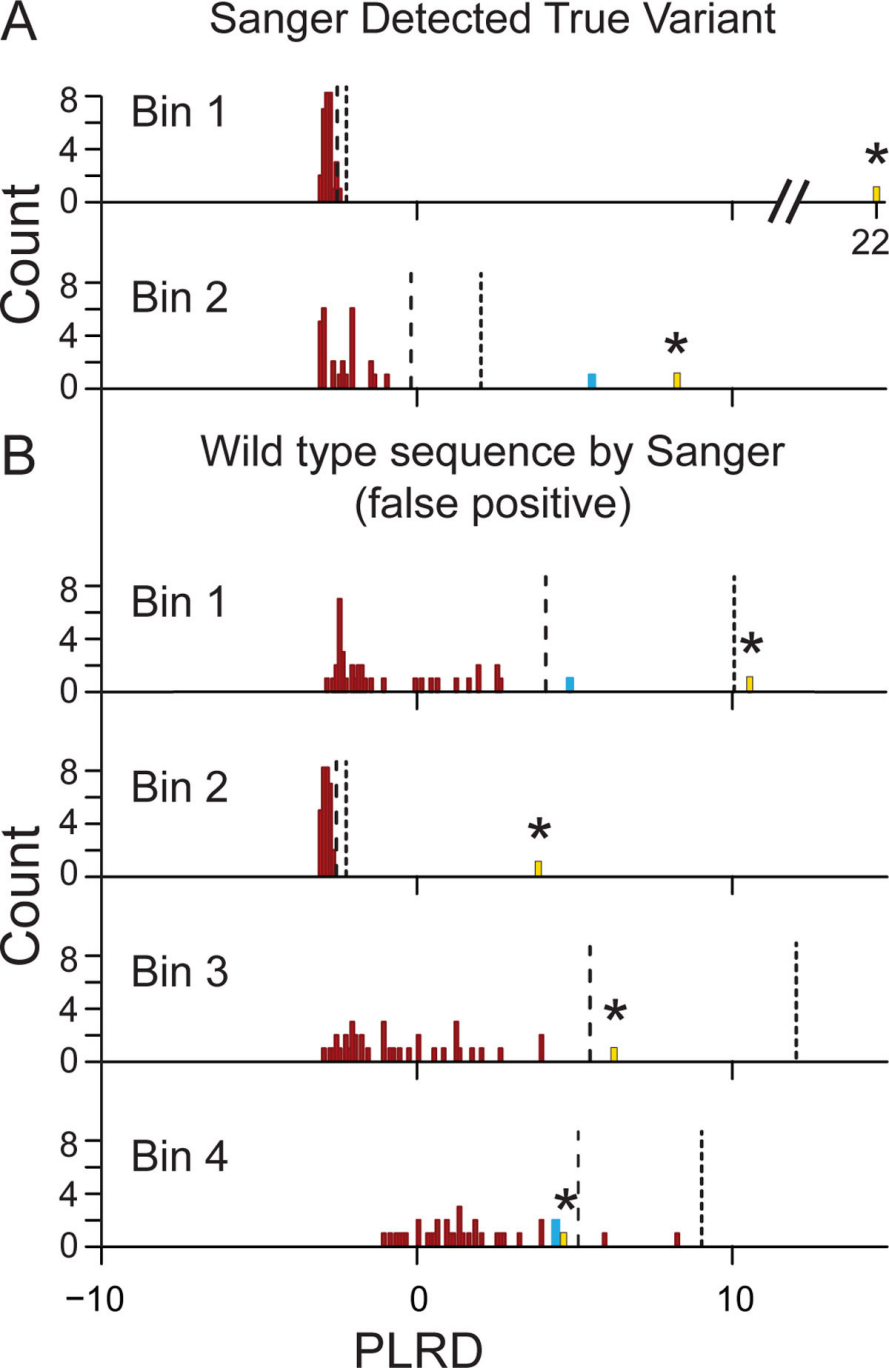
**PLRD histograms by variant position and nucleotide change**. Six example proband variants are shown with data from the proband (gold bar with star), as well as the proband's family and the background samples for the same variant change and position. The PLRD score is plotted versus the sample count. Blue bars indicate for that sample, that variant passed the GATK best practices variant filters. The samples with the red bars did not pass these same filters (wildtype/non-variant) and this variant was not called in their vcf file. The vertical black lines are marking the 3 (dashed line) and 6 (dotted line) standard deviation from the average PLRD score using only the assumed wild type/non-variant PLRD values (red bars, variant was not called). Bin numbers (as described in Methods) are given for each of the proband's variants shown. A) These two variant examples are called as Bin 1 and Bin 2 by the PLRD method and were Sanger verified as true variants. B) Examples of variants from all Bins that were only detected as wild type sequence by Sanger sequencing (false positive variants).

An important feature of the VarBin method is that each variant is evaluated separately, since different positions can have different error rates/propensity due to sequence specific effects [4-6]. The proband's variant PLRD value can be plotted as a histogram with all the background samples' PLRD values for the same variant change and position. The PLRD value spans from likely wild-type/non-variant (approximately -3) to likely true variant (approximately 10 PLRD or greater). As the PLRD distribution broadens for the wild type/non-variants in the background samples, and as the proband PLRD value drops, there is less distinction in the alignment data between the proband variant and the group of wild type/non-variants. This concept was quantified in an empirically derived VarBin method where proband variants were scored as Bin 1 (highly likely a true variant) through Bin 4 (highly likely a false positive) based on the proband PLRD and the wild type/non-variant PLRD distribution median and interquartile distance (IQD). Figure 2 shows example PLRD histograms associated with two Sanger sequence confirmed true variants from Bins 1 and 2 (Figure 2A) and four Sanger confirmed false positive variants from the proband NGS data (Figure 2B). Samples with the variant passing the GATK hard filters are shown in blue, which can include true variants with the potential that some are miscalled, false positive variants. The samples that did not pass the hard filters at the tested variant position are in red, these are termed "wild type/non-variant" and can include samples that are truly wild type at the tested variant position with the potential that some were actually miscalled, false negative variants. Variant PLRD from the proband sample are shown in gold. Variants that were called as homozygous were not considered to be as prone to be false positive variants due to their generally higher total number of variant containing reads and by default were excluded from analysis but were displayed with a PLRD value of zero. These single site histograms in Figure 2 show both typical Bin 1 through 4 examples. In Bin 3 and 4, the PLRD wild type/non-variant (red) distributions are broader and/or closer to the proband variant PLRD for sites and are more likely to be a proband false positive.

### Comparison of VarBin to GATK VQSR

**Figure 3:**
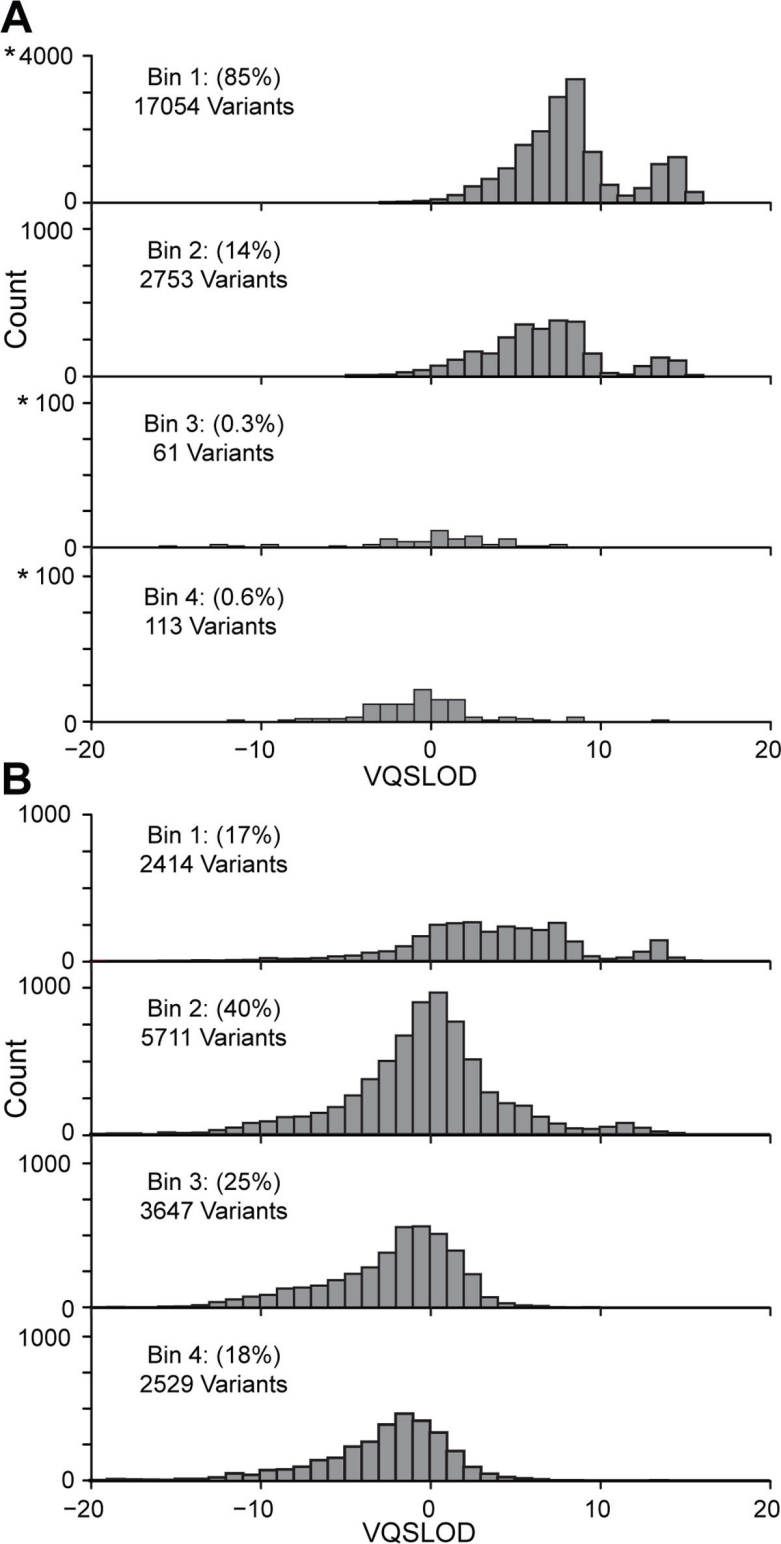
**Comparison of VarBin to VQSLOD**. Variants were separated into Bins using the VarBin method for true variant likelihood (Bin1 most likely true variants, Bin 4 most likely false positive varaints). The Bin groups are displayed in four separate histograms and the total number and percentage of variants in each VarBin Bin group are shown. The corresponding GATK variant quality score recalibration scores (VQSLOD) for each of these Binned variants is plotted on the X-axis versus variant count. Note the starred (*) axis numbers, indicate that the scale is different than the other graphs in the figure. A) A variant set enriched for true variants (approximately 20,000 variants called in the study family proband chromosome one that were also found at between 10 and 20% in 1000 Genomes data set). B) A variant set enriched for false positive variants (approximately 14,500 variants called in the proband's chromosome 1 - 22 that were not found in 1000 Genomes data or in either parent).

The VarBin method's Bin values were compared to the GATK's VQSR score (VQSLOD) using two proband derived variant sets, one set enriched for true variants and one set enriched for false positive variants (same data sets as used in Figure 1). Results for the enriched true variant set (Figure 3A) show that of the approximately 20,000 variant calls, Bins 1 through 4 had 85% (most likely true variants), 14%, 0.2%, and 0.3% variants, respectively. Results for the enriched false positive variant set (Figure 3B) show that of the approximately 14,500 variant calls, Bins 1 through 4 had 17%, 40%, 25%, and 18% variants, respectively. VQSLOD likelihood values for variants in the four separate Bins for both data sets were spread into overlapping distributions with limited correlation between the VarBin method's Bin number and VQSLOD.

### Assessment of VarBin predictions

Sanger sequencing was performed on 98 variants in the proband, the proband family members, and in unrelated families as described in Methods (Table 1). These variants included single nucleotide variants, insertions, deletions, as well as homopolymer and repeat regions. Of the 71 *de novo *variants detected in the proband that were Sanger sequenced, 58 were false positive variants, 4 could not be accurately Sanger sequenced (due to polymerase slipping or they resided in a homologous gene family), 9 were true varaints. Interestingly, 8 of the 9 proband's Sanger confirmed variants that appeared *de novo *were actually present by Sanger sequencing in the proband's family members but these variants were not called variants in their vcf files (non-variant), indicating that the proband wasn't actually *de novo *for those variants and that the VarBin method helped detect these false negative variants. An additional 27 variants (in Bin 1 or 2) in family members or other unrelated individuals were Sanger sequenced to confirm these false negatives and to expand the VarBin study to other families. Combining all Sanger sequencing data for a total of 94 variants (4 were unsuccessful for Sanger), 97%, 30%, 0%, and 0% of the Bin 1, 2, 3, and 4 variant calls, respectively, were true variants (Table 1). The others were Sanger confirmed as wild type sequence and are considered false positive variants. Only one Bin 1 variant was actually a false positive variant, while 10 of 32 Bin 2 variants were true variants.

**Table 1:** Sanger sequencing result is compared with the VarBin variant classification Bin

Bin	Sanger result	Total*	SNV	Indel
Bin 1	true	33	23	10
	false	1	1	-
Bin 2	true	10	8	2
	false	23	21	2
Bin 3	true	-	-	-
	false	16	16	-
Bin 4	true	-	-	-
	false	11	11	-

*98 variants were sequenced in the proband, the proband's family, or other families in the background data sets as stated in the methods. Four of the 98 could not be Sanger sequenced and were excluded from this table (94 total variants shown). SNV, single nucleotide variant; Indel, and insertion or deletion.

### Comparison of proband to background files

In addition to the VarBin method, the individual background files and the proband's family members were used to track the variant frequency and other parameters (such as read coverage, quality information, bias values, number of reads containing variants, tracking low total read coverage depth). The total number of background samples and family members, variant zygosity in the background samples and family members, and which samples had the same variant called as the proband were tracked. This data highlighted how often a variant was called in the background data set, whether the same variant(s) was in an unaffected family member, whether it was in families unaffected by the disease of the proband, and whether other unaffected samples had the same genotype (divergent from the expected Mendelian inheritance). Other tabulated data used for variant prioritization included low variant containing read depth, and low quality scores. These variant calling metrics were collected for all background individuals including proband family members. Because low coverage depth or low quality positions may lead to a missed variant (false negative variant) in a family member which can conceal the true inheritance patterns present at these sites. Using these data in the analysis of the proband's *de novo *variants indicated that approximately 25% were actually in a family member (confirmed by Sanger sequencing) but were missed by the variant calling procedure (false negative varaints). By tracking these parameters in family members as well as use of the VarBin method, many false negative variants were identified within this family.

## Discussion

Variant prioritization of the thousands to millions of variants in a typical gene discovery study is critical to provide focus on the most likely causative variants. As a common step in the analytical process, variants from the affected individual are screened against those found in databases including the 1000 Genomes data and the NHLBI ESP5400 exomes data. Variants are screened by a low minor allele frequency or alternatively all known variants are eliminated. The resulting set of rare or novel variants typically include a much higher fraction of false positive variants than are found in the initial list, since sequence specific error rates are known to be chemistry, instrument and method specific and false positive variants are not commonly expected in the public databases.

The presented VarBin method as well as tracking family member's quality and bias data for the proband's variant facilitates variant prioritization by analyzing predictions of true variant versus false positive variants, the predicted inheritance pattern, and the potential false negative variants. These methods were tested using whole genome sequence data from an example family of four (one affected male proband and the unaffected parents and brother), as well as utilizing a 38 sample background data set.

A number of parameters within GATK or other analysis programs have been developed to evaluate Illumina variants to identify false positive variants. These include quality by depth (QD), raw coverage depth (DP) and a set of alignment bias parameters, but the most common is the variant quality (QUAL) that is derived from a statistical model which incorporates read mapping quality, base quality, base alignment quality, coverage, and variant read percentage [15]. The novel parameter in the VarBin method is the variant-to-non-variant likelihood ratio (PLR), which is related to the variant quality (QUAL) and is derived from the same statistical model. The PLR is a ratio of genotype likelihood (PL) values that are generated by variant detection algorithms found in GATK or Samtools. The PL values compare the three possible genotypes, AA (reference-matching, i.e. wild type sequence), AB (heterozygous variant), or BB (homozygous variant) for a given site and change. Unlike the related variant quality score, QUAL, the PLR equation transitions smoothly from negative to positive as the variant in question moves from inferred false positive variant to inferred true variant. Also, PLR does not include the variant prior probabilities included in the calculation of QUAL.

There is a strong effect of read coverage depth on PLR. As coverage drops, there is less confidence in calling a putative variant as true or false positive, and thus PLR approaches zero. Division of PLR by coverage (PLRD) minimizes the coverage depth dependence. At PLRD equals zero, there is an inferred 50% chance of a true variant with an increasing probability of a true variant as the PLRD value increases. (Of note, homozygous changes were displayed with a PLRD of zero by default, to visually separate them from the heterozygous variant values focused on in this study, as heterozygous variants are more susceptible to the influence of error prone positions potentially causing a false positive variant).

PLRD values were compared between two subsets of variants from the test family's proband sample: a set enriched for true variants and a set enriched for false positive variants. The enriched true variant set demonstrated fairly distinct PLRD value groups for variants that did (called variant in the vcf file) or did not pass (wild type/non-variant) the GATK best practices hard filters for variant calling. In contrast, the enriched false positive variant set demonstrated wild type/non-variants that did not pass filter overlapping into the pass filter variant range of PLRD values (generally greater than zero). This indicates more variability, or noise, in the PLRD for certain variant sites. This increased noise is the result sequencing and alignment errors, as well as error related changes in base and alignment quality scores in the aligned read data used to calculate PLRD. In addition, this PLRD variability was more common for the false positive variants as verified by Sanger sequencing.

Analyzing NGS read data (bam file) using the Broad Institute iGV viewer allowed visual identification of several common features of false positive variants including: fewer variant containing reads compared to wild type reads (low variant allele percentage), variant containing reads were all from one direction (strand bias), the variant was in the same position in all reads (read position bias), the preceding two bases were the same base as the variant change (sequence context), and many background samples had some reads with the variant (variant may or may not have passed GATK filters). In an effort to automate analysis of the information gained from the iGV viewer comparisons (between the family data and multiple background files) to differentiate true variants from the more likely false positive variants, the VarBin method was developed. VarBin analyzes heterozygous variant PLRD distributions at each variant change and position, to compare the proband variant to the family and background data. The chosen PLRD Bin 1 (more likely a true variant) through Bin 4 (more likely a false positive variant) heuristic was informed by visual interpretation of alignment data as an indicator of sequencing or alignment error and then verified by Sanger sequencing data. Median and interquarile distances were used to identify how much the proband's variant differed from the group of background samples PLRD values that were wild type/non-variant.

A typical Sanger confirmed proband variant position commonly had a tight distribution of PLRD values for all the wild type/non-variant background samples, which clustered near -3 PLRD. Commonly, the proband's false positive variants (Sanger verified as wild type) had lower proband PLRD values (usually less than 10 PLRD) as well as an increasingly broad distributions of background sample's wild type/non-variant PLRD values. The higher the proband PLRD and the more separated from the distribution of the non-variant background PLRD values, the more likely the proband variant was anticipated to be a true variant.

To estimate the percentage of variants in Bins 1 through 4 that were true variants, Sanger sequencing was performed on 71 of the proband's *de novo *heterozygous variants. Sanger sequencing for four variants was inconclusive due to Sanger sequencing or PCR issues, like polymerase slipping or difficulty amplifying one gene out of a homologous family of genes, indicating the difficulty of both calling these variants within NGS data and Sanger confirming these variants. An additional 27 Bin 1 and 2 variants were sequenced in the proband's family members or from the background samples. These variants included single nucleotide variants, insertions, deletions, and insertions or deletions at a homopolymer or repeat region. All but one of the Bin 1 variants were Sanger confirmed. The exception variant's PLRD value was near the boundary between Bin 1 and 2 (10 PLRD, 8.28*IQD) and this was the only Bin 1 variant where multiple PLRD values exceeded zero for the background files with no background variants passing filter. This indicates a position prone to false positive variants. But since the proband's variant had a high probability to be true, greater than 8.28*IQD from the mean and >10 PLRD, it was included in Bin 1. This example highlights that VarBin allows for Bin 1 variants even at sites prone to false positive variants (broad distribution of wild-type/non-variant background PLRD values) if the variant PLRD value is adequately separated from the background non-variant PLRD values. Thus, VarBin is an alternative to other methods that propose rejection of all variants in false positive prone regions or, reject variants at specific site/nucleotide change combinations [22,23].

All Bin 3 or 4 variants were found to be false positives variants by Sanger sequencing, indicating that Bin 3 and 4 may be combined into one bin. Sanger sequencing also indicated that 30% of variants in Bin 2 were true variants. The majority of the true Bin 2 variants present in other family members were in Bin 1 for the proband (8/9 "*de novo*" proband variants were also in one of the parents). These true variants were originally called "wildtype/non-variant", so they were false negative variants. The chosen GATK hard filters eliminated these variants, while the VarBin method had put them in Bin 2, which would be prioritized for further analysis since ~30% of variants in Bin 2 are true variants. This indicates the VarBin method's potential for detection of false negatives within a family or potentially within one sample. The majority of false positive Bin 2 variants and the only Bin 1 false variant had a polymer motif leading up to the change (e.g. GGT to GGG). This motif is well known to lead to Illumina sequence specific increased errors [4,6], and may help further classify certain variants within Bin 2 with these motifs as potentially false positive variants.

The VarBin method differs significantly from other commonly used tools for false positive likelihood determination such as the GATK variant quality score recalibration (VQSR) [14]. VQSR uses a training set of variants across the genome in the sample of interest to train a Gaussian mixture model for true variant detection based on several variant parameters of quality and bias. Unlike VQSR, the presented VarBin method does not depend on a model generalized over a broad range of many variants and variant contexts. Instead this method evaluates each variant site separately and uses multiple, locally sequenced background samples to increase variant likelihood information about each specific variant change and position, even if the proband variant of interest was not called a variant within a background sample (wild type/non-variant). Results from the VarBin method were compared to Phred-scaled VQSR likelihood ratios of a true-to-false variant (VQSLOD) results for a set of proband variants that were enriched for true variants or enriched for false positive variants. For the enriched for true variant data, the variants primarily scored as Bin 1 (85%) with only a small number scored as Bin 3 or 4 (1%). For the enriched false positive variant data set the majority of variants fell in Bin 2 followed by 3 and 4. Each Bin consisted of a relatively wide range of VQSLOD values, indicating limited correlation of VarBin and VQSR methods for detection of false positive variants.

Bin 1 variants are likely true variants due to the proband variant PLRD score's larger separation from the distribution of background samples' wild type/non-variant PLRD scores. In Bin 2, variants were separate enough from the non-variant background samples to have a probability of being true variants, but usually had one or more factor(s) that could indicate a false positive variant, such as read strand bias, read position bias, low quality, and low count for variant containing reads. The median VQSLOD value for the Bin 1 variants (8.2) corresponded to an expected 87% true variant estimate. The Sanger sequencing for this data set indicates a 93% true variant result for Bin 1, but only 30% true variants in Bin 2. All Bin 3 and 4 proband variants PLRD values were near or within the range of background samples non-variants' PLRD values, indicating that these are the most likely false positive variants. All variants in Bins 3 and 4 were confirmed to be wild type by Sanger sequence, and therefore false positive NGS detected variants. Thus, Bin 3 and Bin 4 could possibly be merged into a single Bin of false positive variants. The VQSLOD median value for Bin 4 variants (-7.5) corresponded to an expected 18% true variants. The PLRD-based VarBin method presented in this study appears to provide useful, improved segregation of false positive variants (Bin 3 and 4) and true variant (Bin 1) calls, with Bin 2 being uncertain (containing true and false positive variants). This indicates that only Bin 1 and a portion of Bin 2 variants would be prioritized for further analysis in gene discovery studies.

## Conclusions

VarBin was created to classify false positive variants from true variants in Illumina data sets. VarBin was also created to automate the manual processes often used to analyze NGS data for visual indicators of false positive variants. The VarBin method accurately Binned variants into different levels of true variant likelihood, as confirmed by Sanger sequencing, where Bin 1 is most likely true, Bin 3 and 4 were false positive variants, and Bin 2 was uncertain (70% false positive). In addition, Bin 2 variants were commonly true if the same variant was seen in Bin 1 for a family member, highlighting the usefulness of family based data in false positive variant identification. Of note, family data is not required for VarBin and VarBin can classify insertions, frame shifts and deletions, as well as single nucleotide variants. The PLRD and variant filter parameter information for these "non-variants" is useful for false negative variant detection in family member samples used as background samples.

Future work on the VarBin method will focus on differentiating true variants from false positive variants within Bin 2, and converting the VarBin result from a discrete Bin to a continuous parameter. Additional efforts will focus on using VarBin to identify false negative variants ("non-variants" that did not pass filter but were Sanger verified as true variants) in Bin 1 or 2. The VarBin method could also be incorporated into the GATK VQSR method for a potentially more accurate false positive variant detection. PLRD values for background files can also be pre-calculated to speed analysis. Also, we will explore using the VarBin method for other NGS platforms and library preparation methods (such as for different targeted capture methods). In conclusion, VarBin improves the accuracy of classifying true variants and false positives variants within Illumina NGS data based on the comparison of the VarBin method to VQSR and VarBin predictions being verified by the Sanger sequencing results.

## Competing interests

The authors declare that they have no competing interests.

## Authors' contributions

JD carried out bioinformatic analysis and jointly drafted the manuscript. RLM performed sequencing, sequencing data analysis, and jointly drafted the manuscript. EMC participated in the sequencing and sequencing data analysis. KCM carried out Sanger sequencing. KVV participated in the design and coordination of the study and helped to draft the manuscript.

## Declarations

Funding for research and publication of this article came solely from the ARUP Institute for Clinical & Experimental Pathology®, Salt Lake City, Utah.

This article has been published as part of BMC Bioinformatics Volume 14 Supplement 13, 2013: Selected articles from the 9th Annual Biotechnology and Bioinformatics Symposium (BIOT 2012). The full contents of the supplement are available online at http://www.biomedcentral.com/bmcbioinformatics/supplements/14/S13

## References

[B1] Coonrod EM Durtschi JD Margraf RL Voelkerding KV Developing Genome and Exome Sequencing for Candidate Gene Identification in Inherited Disorders Archives of pathology & laboratory medicine 2012 22770468 10.5858/arpa.2012-0107-RA

[B2] Ledergerber C Dessimoz C Base-calling for next-generation sequencing platforms Brief Bioinform 2011 12 5 489 497 10.1093/bib/bbq077 21245079 PMC3178052

[B3] Luedtke A Powers S Petersen A Sitarik A Bekmetjev A Tintle NL Evaluating methods for the analysis of rare variants in sequence data BMC Proc 2011 5 Suppl 9 S119 10.1186/1753-6561-5-S9-S119 22373354 PMC3287843

[B4] Abnizova I Leonard S Skelly T Brown A Jackson D Gourtovaia M Qi G Te Boekhorst R Faruque N Lewis K Analysis of context-dependent errors for illumina sequencing J Bioinform Comput Biol 2012 10 2 1241005 10.1142/S0219720012410053 22809341

[B5] Flaherty P Natsoulis G Muralidharan O Winters M Buenrostro J Bell J Brown S Holodniy M Zhang N Ji HP Ultrasensitive detection of rare mutations using next-generation targeted resequencing Nucleic acids research 2012 40 1 e2 10.1093/nar/gkr861 22013163 PMC3245950

[B6] Meacham F Boffelli D Dhahbi J Martin DI Singer M Pachter L Identification and correction of systematic error in high-throughput sequence data BMC bioinformatics 2011 12 451 10.1186/1471-2105-12-451 22099972 PMC3295828

[B7] Minoche AE Dohm JC Himmelbauer H Evaluation of genomic high-throughput sequencing data generated on Illumina HiSeq and genome analyzer systems Genome Biol 2011 12 11 R112 10.1186/gb-2011-12-11-r112 22067484 PMC3334598

[B8] Lee H Schatz MC Genomic dark matter: the reliability of short read mapping illustrated by the genome mappability score Bioinformatics (Oxford, England) 2012 28 16 2097 2105 10.1093/bioinformatics/bts330 PMC3413383 22668792

[B9] Treangen TJ Salzberg SL Repetitive DNA and next-generation sequencing: computational challenges and solutions Nat Rev Genet 2012 13 1 36 46 22124482 10.1038/nrg3117 PMC3324860

[B10] Bansal V Harismendy O Tewhey R Murray SS Schork NJ Topol EJ Frazer KA Accurate detection and genotyping of SNPs utilizing population sequencing data Genome Res 20 4 537 545 20150320 10.1101/gr.100040.109 PMC2847757

[B11] Margraf RL Durtschi JD Dames S Pattison DC Stephens JE Mao R Voelkerding KV Multi-sample pooling and illumina genome analyzer sequencing methods to determine gene sequence variation for database development Journal of biomolecular techniques: JBT 2010 21 3 126 140 20808642 PMC2922832

[B12] Margraf RL Durtschi JD Dames S Pattison DC Stephens JE Voelkerding KV Variant identification in multi-sample pools by illumina genome analyzer sequencing Journal of biomolecular techniques: JBT 2011 22 2 74 84 21738440 PMC3121147

[B13] Muralidharan O Natsoulis G Bell J Newburger D Xu H Kela I Ji H Zhang N A cross-sample statistical model for SNP detection in short-read sequencing data Nucleic acids research 2012 40 1 e5 10.1093/nar/gkr851 22064853 PMC3245949

[B14] DePristo MA Banks E Poplin R Garimella KV Maguire JR Hartl C Philippakis AA del Angel G Rivas MA Hanna M A framework for variation discovery and genotyping using next-generation DNA sequencing data Nature genetics 2011 43 5 491 498 10.1038/ng.806 21478889 PMC3083463

[B15] Li H A statistical framework for SNP calling, mutation discovery, association mapping and population genetical parameter estimation from sequencing data Bioinformatics (Oxford, England) 2011 27 21 2987 2993 10.1093/bioinformatics/btr509 PMC3198575 21903627

[B16] Li H Improving SNP discovery by base alignment quality Bioinformatics (Oxford, England) 2011 27 8 1157 1158 10.1093/bioinformatics/btr076 PMC3072548 21320865

[B17] Ho MR Tsai KW Chen CH Lin WC dbDNV: a resource of duplicated gene nucleotide variants in human genome Nucleic acids research 2011 39 Database D920 925 10.1093/nar/gkq1197 21097891 PMC3013738

[B18] Simola DF Kim J Sniper: improved SNP discovery by multiply mapping deep sequenced reads Genome Biol 2011 12 6 R55 10.1186/gb-2011-12-6-r55 21689413 PMC3218843

[B19] Shen Y Wan Z Coarfa C Drabek R Chen L Ostrowski EA Liu Y Weinstock GM Wheeler DA Gibbs RA A SNP discovery method to assess variant allele probability from next-generation resequencing data Genome Res 2010 20 2 273 280 10.1101/gr.096388.109 20019143 PMC2813483

[B20] McKenna A Hanna M Banks E Sivachenko A Cibulskis K Kernytsky A Garimella K Altshuler D Gabriel S Daly M The Genome Analysis Toolkit: a MapReduce framework for analyzing next-generation DNA sequencing data Genome Res 2010 20 9 1297 1303 10.1101/gr.107524.110 20644199 PMC2928508

[B21] Thorvaldsdottir H RoBinson JT Mesirov JP Integrative Genomics Viewer (IGV): high-performance genomics data visualization and exploration Brief Bioinform 2012 10.1093/bib/bbs017 PMC3603213 22517427

[B22] Adams DR Sincan M Fuentes Fajardo K Mullikin JC Pierson TM Toro C Boerkoel CF Tifft CJ Gahl WA Markello TC Analysis of DNA sequence variants detected by high-throughput sequencing Hum Mutat 2012 33 4 599 608 10.1002/humu.22035 22290882 PMC3959770

[B23] Fuentes Fajardo KV Adams D Program NCS Mason CE Sincan M Tifft C Toro C Boerkoel CF Gahl W Markello T Detecting false-positive signals in exome sequencing Hum Mutat 2012 33 4 609 613 10.1002/humu.22033 22294350 PMC3302978

